# Magnetization transfer imaging of ovarian cancer: initial experiences of correlation with tissue cellularity and changes following neoadjuvant chemotherapy

**DOI:** 10.1259/bjro.20210078

**Published:** 2022-05-02

**Authors:** Surrin S Deen, Mary A McLean, Andrew B Gill, Robin A F Crawford, John Latimer, Peter Baldwin, Helena M Earl, Christine A Parkinson, Sarah Smith, Charlotte Hodgkin, Mercedes Jimenez-Linan, Cara R Brodie, Ilse Patterson, Helen C Addley, Susan J Freeman, Penelope M Moyle, Martin J Graves, Evis Sala, James D Brenton, Ferdia A Gallagher

**Affiliations:** ^1^ Department of Radiology, Cambridge University Hospitals NHS Foundation Trust, Addenbrooke’s Hospital, Cambridge, United Kingdom, CB2 0QQ; ^2^ Cancer Research UK Cambridge Institute, University of Cambridge, Cambridge, United Kingdom, CB2 0RE; ^3^ Department of Radiology, Box 218, University of Cambridge, Cambridge, United Kingdom, CB2 0QQ; ^4^ Cambridge University Hospitals NHS Foundation Trust, Addenbrooke’s Hospital, Cambridge, United Kingdom, CB2 0QQ; ^5^ Department of Oncology, Cambridge University Hospitals NHS Foundation Trust, Addenbrooke’s Hospital, Cambridge, United Kingdom, CB2 0QQ; ^6^ Department of Pathology, Cambridge University Hospitals NHS Foundation Trust, Addenbrooke’s Hospital, Cambridge, United Kingdom, CB2 0QQ

## Abstract

**Objectives:**

To investigate the relationship between magnetization transfer (MT) imaging and tissue macromolecules in high-grade serous ovarian cancer (HGSOC) and whether MT ratio (MTR) changes following neoadjuvant chemotherapy (NACT).

**Methods:**

This was a prospective observational study. 12 HGSOC patients were imaged before treatment. MTR was compared to quantified tissue histology and immunohistochemistry. For a subset of patients (*n* = 5), MT imaging was repeated after NACT. The Shapiro–Wilk test was used to assess for normality of data and Spearman’s rank-order or Pearson’s correlation tests were then used to compare MTR with tissue quantifications. The Wilcoxon signed-rank test was used to assess for changes in MTR after treatment.

**Results:**

Treatment-naïve tumour MTR was 21.9 ± 3.1% (mean ± S.D.). MTR had a positive correlation with cellularity, rho = 0.56 (*p* < 0.05) and a negative correlation with tumour volume, ρ = −0.72 (*p* = 0.01). MTR did not correlate with the extracellular proteins, collagen IV or laminin (*p* = 0.40 and *p* = 0.90). For those patients imaged before and after NACT, an increase in MTR was observed in each case with mean MTR 20.6 ± 3.1% (median 21.1) pre-treatment and 25.6 ± 3.4% (median 26.5) post-treatment (*p* = 0.06).

**Conclusion:**

In treatment-naïve HGSOC, MTR is associated with cellularity, possibly reflecting intracellular macromolecular concentration. MT may also detect the HGSOC response to NACT, however larger studies are required to validate this finding.

**Advances in knowledge:**

MTR in HGSOC is influenced by cellularity. This may be applied to assess for cell changes following treatment.

## Introduction

Ovarian cancer is the gynaecological malignancy with the highest mortality worldwide and high-grade serous ovarian cancer (HGSOC) is the most common and malignant form of ovarian cancer, making it the most clinically relevant form of ovarian cancer to investigate.^1^ Treatment options for HGSOC remain limited but recently new therapies such as PARP (poly-ADP-ribose polymerase) inhibitors,^2^ immune checkpoint inhibitors^3^ and VEGF (vascular endothelial growth factor) inhibitors^4^ have shown promise for improving the outlook of the disease. With new treatment options, there also comes a need to improve the detection of response to guide clinical management decisions, particularly for targeted therapies where there may not be a significant alteration in tumour size, and for combination therapies, where there may be multiple biological changes occurring simultaneously. Magnetization transfer (MT) imaging is a fast and simple technique to implement in a clinical setting^5–7^ whose relationship with tumour cellularity, major extracellular macromolecules and potential to detect a response to standard-of-care neoadjuvant chemotherapy (NACT) we have investigated in this study.

The majority of water molecules *in vivo* are freely moving, but a minority are bound to tissue macromolecules. This bound water is loosely attached to charged macromolecules forming hydration layers.^8^ The local magnetic fields of the charged macromolecules to which bound water molecules are attached produce a broadening in the range of frequencies at which magnetization can be absorbed. Bound water magnetization may transfer to the free water pool, thereby saturating the free water molecule pool and reducing its signal.^9^ In MT imaging, an off-resonance pulse is used to preferentially saturate bound water molecules and the resultant reduction in free water signal is quantified via the magnetization transfer ratio (MTR), which therefore functions as an indirect metric of macromolecular binding.^10,11^

Collagen is the most abundant protein in the human body^12^ and bound water plays a key role in maintaining its conformation.^13^ Preclinical experiments both *in vitro* and *in vivo* have demonstrated that MT imaging can detect denaturation of collagen.^14,15^ Changes in collagen cross-linking in the extracellular matrix (ECM) are known to occur in many cancers^16,17^ and a recent preclinical study showed that MT reports on fibrosis and total collagen deposition in an orthotopic model of ovarian cancer.^18^ This previous study however did not differentiate between intracellular and extracellular collagen. Other studies have shown that a collagen-remodelling gene signature is associated with poor outcome and metastases in HGSOC,^19^ and that in NACT treatment of ovarian cancer, the upregulation of collagen expression is related to increased resistance to therapy and the inhibition of apoptosis.^20–22^ These suggest that MTR may change in response to treatment as chemotherapy either selects for more resistant ovarian cancer cell lines or causes an inflammatory tissue reaction leading to fibrosis.

The application of MT to clinical oncology has previously been investigated in a variety of tumours including breast and prostate cancer,^23,24^ and some clinical potential has already been demonstrated. For example, MT has been shown to detect microstructural differences between pathologic and normal pancreatic tissue^25^ and to provide information on the extracellular matrix structure of some cancers.^26–28^ We have recently also shown that there is a higher MTR in prostate cancer compared to benign tissue.^24^ The specific macromolecules that determine the measured MT signal in cancer, as well as the location of these macromolecules within the tumour microenvironment, is still unknown. A better understanding of the biological basis for the MT signal could provide non-invasive information on microscopic changes across an entire tumour volume during therapy that can complement focused histological analysis and provide an alternative source of information in patients who may be unfit or otherwise inappropriate to undergo biopsy.

The primary aim of this study was to explore the relationship between MT imaging and microstructural tumour features in patients with HGSOC through quantitative histopathological analysis of cellularity and macromolecular concentrations to gain insight on the biological origins of the major contributors to MTR in HGSOC. As an exploratory end point to this study, a subset of patients was also re-imaged after three cycles of NACT to investigate whether the known microstructural changes that occur in response to chemotherapy, such as increased fibrosis and collagen deposition, could lead to alterations in MTR that are detectable with a clinically translatable imaging technique.

## Methods and materials

### Study design

**Figure 1.**
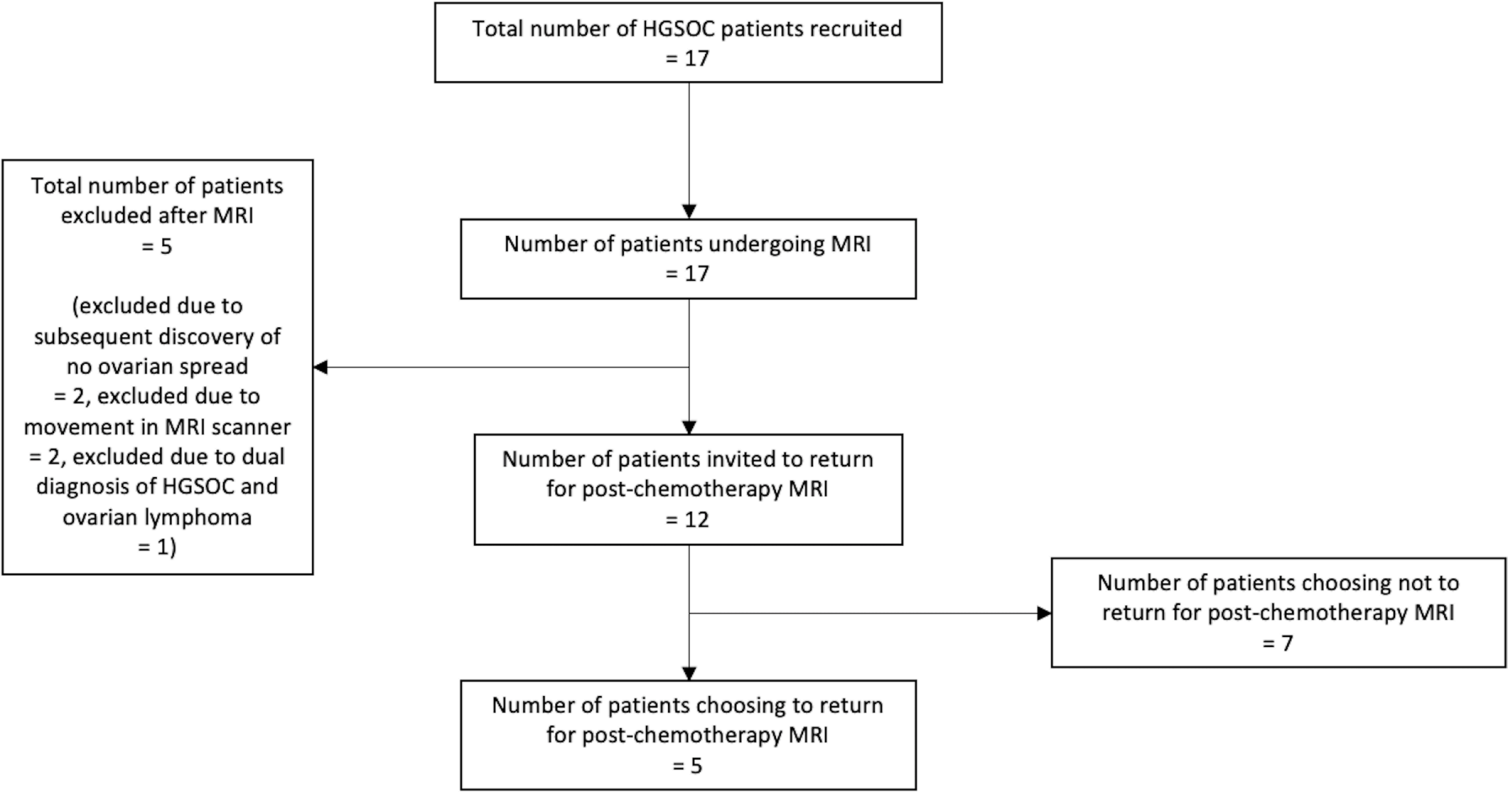
Flowchart of patient recruitment and progression through study.

This was a single centre prospective observational study. Consecutively presenting patients referred with a new histologically confirmed diagnosis of HGSOC from a tissue sample taken from the ovary or peritoneum, no previous chemotherapy treatment and no contraindications to MRI were invited to take part. HGSOC originates from epithelial fallopian tube cells and spreads to the ovaries and peritoneum. Ovarian and peritoneal lesions in HGSOC are very different histopathologically and microstructurally, with peritoneal lesions having a much higher fat content. Therefore, to maintain tissue homogeneity in our study population, only solid ovarian lesions were assessed and any HGSOC patients found after recruitment to suffer only from peritoneal spread with no ovarian disease were excluded. Patients with previous exposure to chemotherapy and those who were unsuitable for MRI, *e.g.* due to metal implants, extreme obesity or inability to remain still were also excluded. Recruitment took place over 13 months from August 2016 to August 2017. After recruitment of the last patient, a three cycle chemotherapy course with 3 months between each cycle had to be completed by the last patient before data analysis could begin. A flowchart of patient recruitment and progression through the study is shown in Figure 1. Institutional Review Board (IRB) approval was obtained (Reference: 15/EE/0378), and the study was registered on the public database ClinicalTrials.gov (Identifier: NCT03526809). All study related procedures were performed with the written informed consent of participants and in accordance with the ethical principles outlined in the Declaration of Helsinki.

17 HGSOC patients were recruited, of which five were non-evaluable. Of the non-evaluable patients, two could not remain still and movement between the MT_off_ and MT_on_ imaging caused significant artefact in the calculated MTR maps that rendered the measurements unreliable, two had only peritoneal disease and no spread within the ovaries, and one was diagnosed with both HGSOC and lymphoma, which would have influenced the imaging-histology comparison. Of the 12 remaining patients, all were offered the free choice to return for a repeat MRI scan after three cycles of standard-of-care NACT treatment with carboplatin and paclitaxel of these, five patients chose to have post-NACT treatment imaging.

### Imaging protocols and region of interest analysis

MRI, including *T*_2_-weighted and MT imaging, was performed on a Discovery 3T MR750 system (GE Healthcare, Waukesha WI). 20 mg of hyoscine butylbromide (buscopan) was given to participants intravenously to reduce bowel motion 10 min before scanning. MT imaging was performed with the following scan parameters: TR (repetition time)=24 ms, TE (echo time)=2.8 ms, flip angle = 5°, slice thickness = 5 mm, matrix = 256 x 192, FoV (Field of View)=34.0 x 34.0 cm, NEX (number of excitations)=1. The MT_on_ acquisition included an 8 ms Fermi saturation pulse with nominal flip angle 360° and frequency offset = 2200 Hz. *T*_2_ weighted imaging was performed with a fast spin echo sequence with TR = 4000 ms, TE = 91.1 ms, flip angle = 90°, slice thickness = 6 mm, FoV 34.0 × 29.9 cm and NEX = 8.

MTRs in percentages were calculated from the MT_on_ and MT_off_ images according to Equation (1) shown below, where MT_on_ and MT_off_ represent the signal intensities of the MT_on_ and MT_off_ images respectively:

MTR=(MToff−MTon)/MToff×100

The post-treatment imaging of the five patients was performed at 1to 7 days (median 3 days) after the end of the third cycle of standard-of-care neoadjuvant chemotherapy.

Regions of interest (ROIs) were selected on the *T*_2_ weighted images by a radiologist with nine years of experience as an attending radiologist, who was blinded to the MT images and tissue immunohistochemistry results. ROIs were drawn around all the slices comprising the solid HGSOC cancer volumes located in the ovaries with OsiriX (v. 3.8.1, Pixmeo, Geneva, Switzerland), non-solid components of the lesions were excluded. The ROIs were imported from the *T*_2_ weighted images onto the MTR maps that were aligned to the *T*_2_ weighted images in Osirix and mean MTRs for the cancer volumes were calculated. Intra- and interobserver variability were assessed with additional ROIs drawn by a radiology resident with four years of further experience as a radiology researcher in oncological and gynaecological MRI.

### Tissue analysis

Tumour samples were collected at diagnosis from patients before the start of any treatment either by percutaneous ultrasound-guided biopsy or a surgical procedure depending on lesion location and accessibility. The biopsied tissue was fixed in formalin and embedded into paraffin blocks. Sections 3 µm thick were cut from the blocks and the slides were stained using Leica’s Polymer Refine Detection System automated Bond platform (DS9800, Leica Biosystems, Germany). Bright-field scanning with an Aperio AT2 scanner (Leica Biosystems, Germany) was carried out to digitise the slides, to automatically identify the tissue boundaries and to calculate the total tissue area. The automatically located tissue boundaries were also visually reviewed by a human operator in each case to verify accurate placement by the software.

Tumour cell densities were calculated from fixed tissue on representative slides stained with hematoxylin to identify the nuclei for the cell counting. The cell count was performed using the multiplex IHC V1.2 module of Halo histology image analysis software (Indica labs, Albuquerque, NM, v. 2.1.1637.11). Cell count per unit tissue area was assumed to be representative of the intracellular volume fraction of the tissue and therefore proportional to the total intracellular macromolecular concentration of the tissue.

The extracellular macromolecular concentrations of the tumour samples were estimated by semi-automated immunohistochemistry (IHC) quantification of two different extracellular proteins: collagen type IV and laminin. Collagen type IV and laminin are almost entirely isolated to the extracellular space, in particular to the basal lamina layer of the basement membrane (BM).^29^ Given their spatial distribution, tissue collagen IV and laminin concentrations were assumed to change proportionally to other more abundant extracellular macromolecules, and therefore to be surrogate markers of the total macromolecular concentrations of the tissue samples for the purposes of the correlation analyses performed in this study. Collagen IV was stained with collagen type IV mouse antibody, (M0785, Dako, Santa Clara, CA), with a dilution of 1:50 and retrieval using proteinase K, 20’. Laminin was stained with laminin rabbit antibody, (L9393 Sigma-Aldrich, Missouri) using a dilution of 1:1000 and retrieval with sodium citrate, 5’ and proteinase K, 5’. The quantification of the collagen IV and laminin-stained tissue were also performed using the multiplex IHC V1.2 module of Halo histology image analysis software (Indica labs, Albuquerque, NM, v. 2.1.1637.11). For both collagen IV and laminin, the slide areas that stained with a greater than 0.355 optical density (OD) were considered positive for the respective molecule. The percentages of areas positive for collagen and laminin were calculated by dividing the positive tissue stain area for each molecule by the total tissue area of the slide. The operator of the IHC analytic software was kept blind to the MT analysis results.

### Statistical methods

All statistical analysis was performed using SPSS (IBM Corp. Released 2017. IBM SPSS Statistics for Macintosh, v. 25.0. Armonk, NY: IBM Corp) and a *p-*value of 0.05 was used as the threshold for significance. Intra- and interobserver variability were assessed with the intraclass correlation coefficient (ICC). Immunohistochemistry quantification and MT data were first assessed for normality using the Shapiro–Wilk test and then analysed using either Pearson’s correlation if normally distributed or Spearman’s rank order correlation if not normally distributed. The change in mean MTR after treatment was assessed using the Wilcoxon signed-rank test for non-parametric paired data.

## Results

### Patients and imaging

**Figure 2.**
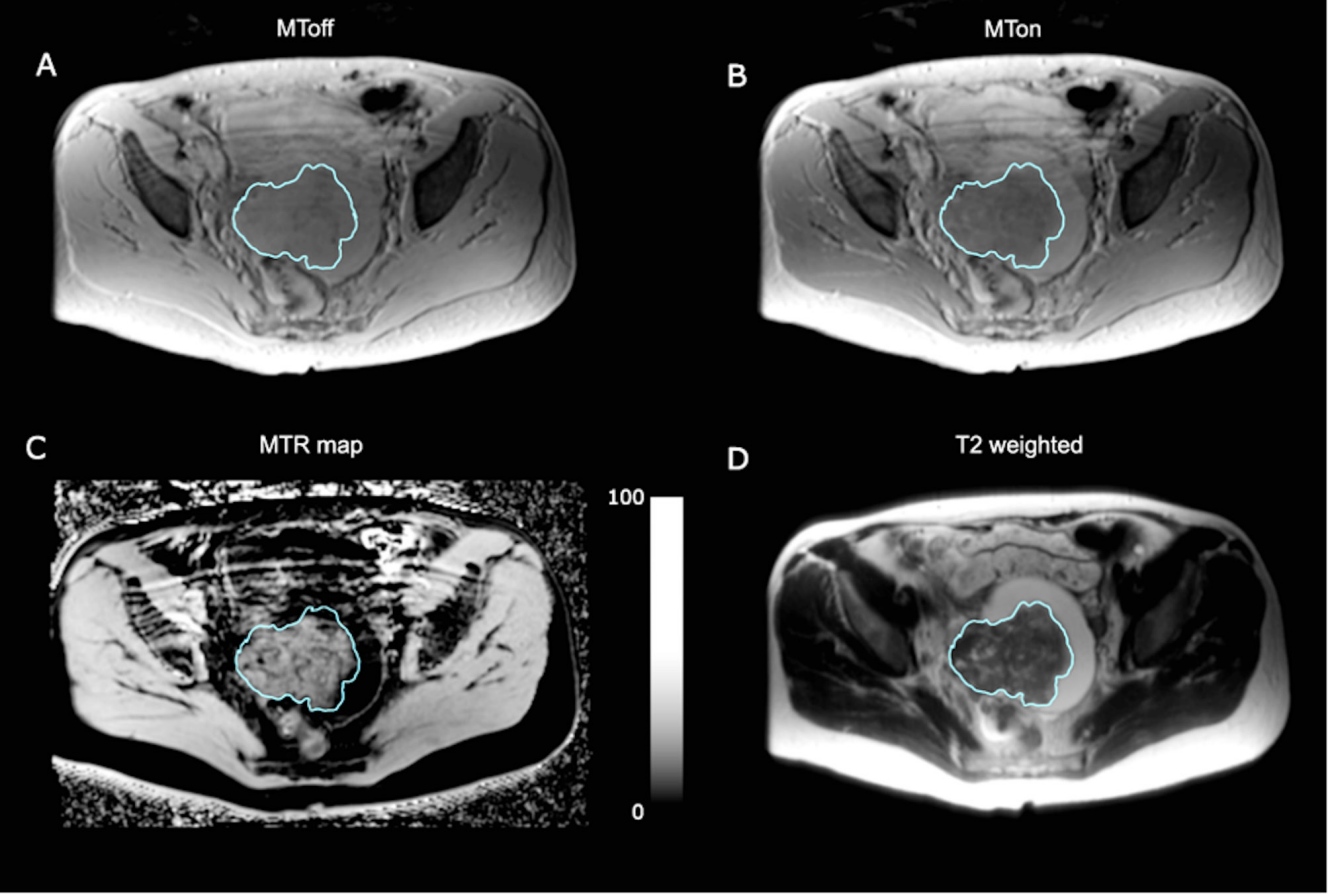
Example axial images from a 63-year-old participant. The tumour is outlined in blue. (**A**) MT_off_; (**B**) MT_on_; (**C**) MTR map, scale bar represents MTR in percent; (**D**) *T*_2_ weighted image. MT = magnetization transfer; MTR = magnetization transfer ratio.

Median age of the evaluated participants was 69 years (range 51 to 81) and for patients who had repeat imaging the median age was 63 years (range 60 to 72). Patient characteristics are summarised in Table 1. Figure 2 shows an MT imaging example from a 63-year-old HGSOC patient. The mean MTR for HGSOC lesions across all participants before NACT treatment was 21.9 ± 3.1% (mean ± SD). Mean lesion volume was 93.2 ± 89.2 cm^3^.

**Table 1.** Population demographics of patients recruited to the study

Feature	Value
Number of evaluated patients (number evaluated post-treatment)	12 (5)
Median age (range) in years, for all evaluated patients	69 (51 to 81)
Median age (range) in years, for post-treatment imaging patients	63 (60 to 72)
**FIGO stage**	**Number of patients**
I	0
II	1
III	8
IV	3
**Volume of lesion (cm^3^)**	**Number of patients**
0–50	4
50–100	3
>100	5

FIGO = Fédération Internationale de Gynécologie et d'Obstétrique, CA 125 = cancer antigen 125.

### Intraobserver and interobserver variability

For the initial MT imaging of the 12 evaluated patients, the interobserver ICC was 0.964 with a 95% confidence interval of 0.881 to 0.990, and the intraobserver ICC was 0.981 with a 95% confidence interval of 0.936 to 0.995. For the five post-treatment cases, the interobserver ICC for post-treatment MT imaging was 0.966 with a 95% confidence interval of 0.715 to 0.996 and the intraobserver ICC was 0.971 with a 95% confidence interval of 0.859 to 0.998.

### Tissue immunohistochemistry

**Figure 3.**
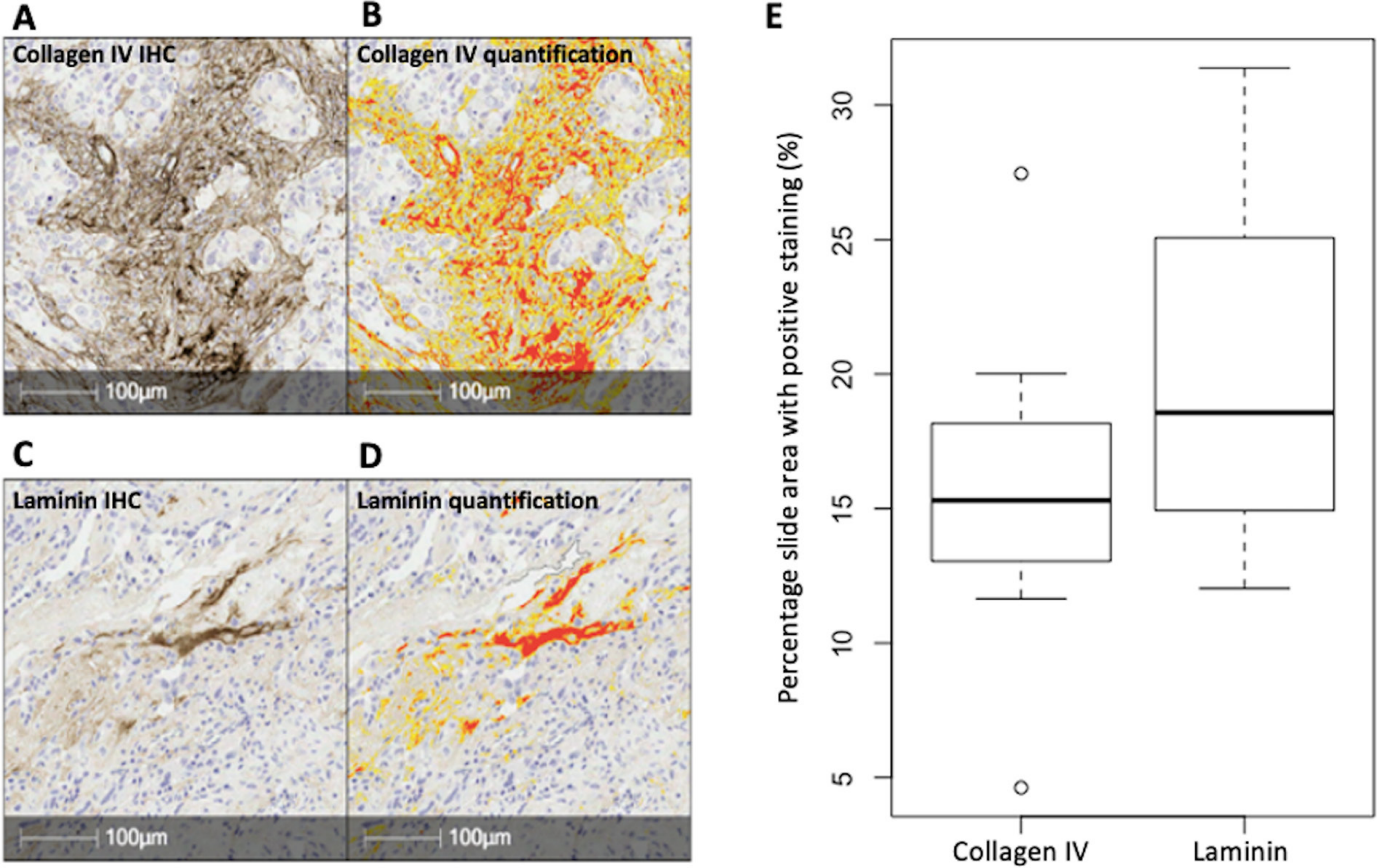
High-grade serous ovarian cancer histology and IHC from a 63-year-old patient at 20x magnification. (**A**) Collagen IV IHC: the brown stain represents positive expression, and the blue stain represents the hematoxylin background counterstain. (**B**) Automated segmentation of the collagen IV staining: yellow segmentation represents staining with OD 0.270–0.355 and red segmentation represents staining with OD >0.355. (**C**) Laminin IHC: the brown stain represents positive expression, and the blue stain represents the hematoxylin background counterstain. (**D**) Automated segmentation of the laminin staining: yellow segmentation represents staining with OD = 0.270–0.355 and red segmentation represents staining with OD >0.355. (**E**) Box-plots showing the distribution of percentage slide area with positive collagen IV and laminin staining. IHC = immunohistochemistry; OD = optical density.

Representative examples of the collagen IV and laminin immunohistochemical staining for a 63-year-old patient are given in Figure 3A and C respectively. Figure 3B and D show the automated segmentation of the IHC staining during quantification. On visual inspection, the collagen IV and laminin were both localised to the extracellular space as expected. Figure 3E depicts boxplots of the percentage slide staining of collagen IV and laminin for all evaluated cases combined: the mean percentage positive tissue area was 15.7 ± 5.5% for collagen IV and 20.0 ± 6.3% for laminin. The mean number of cells per unit slide area was 6.88 ± 3.19 x 10^−3^ cells/μm^2^.

### Correlation of cellularity, tumour volume and IHC with MTR

**Figure 4.**
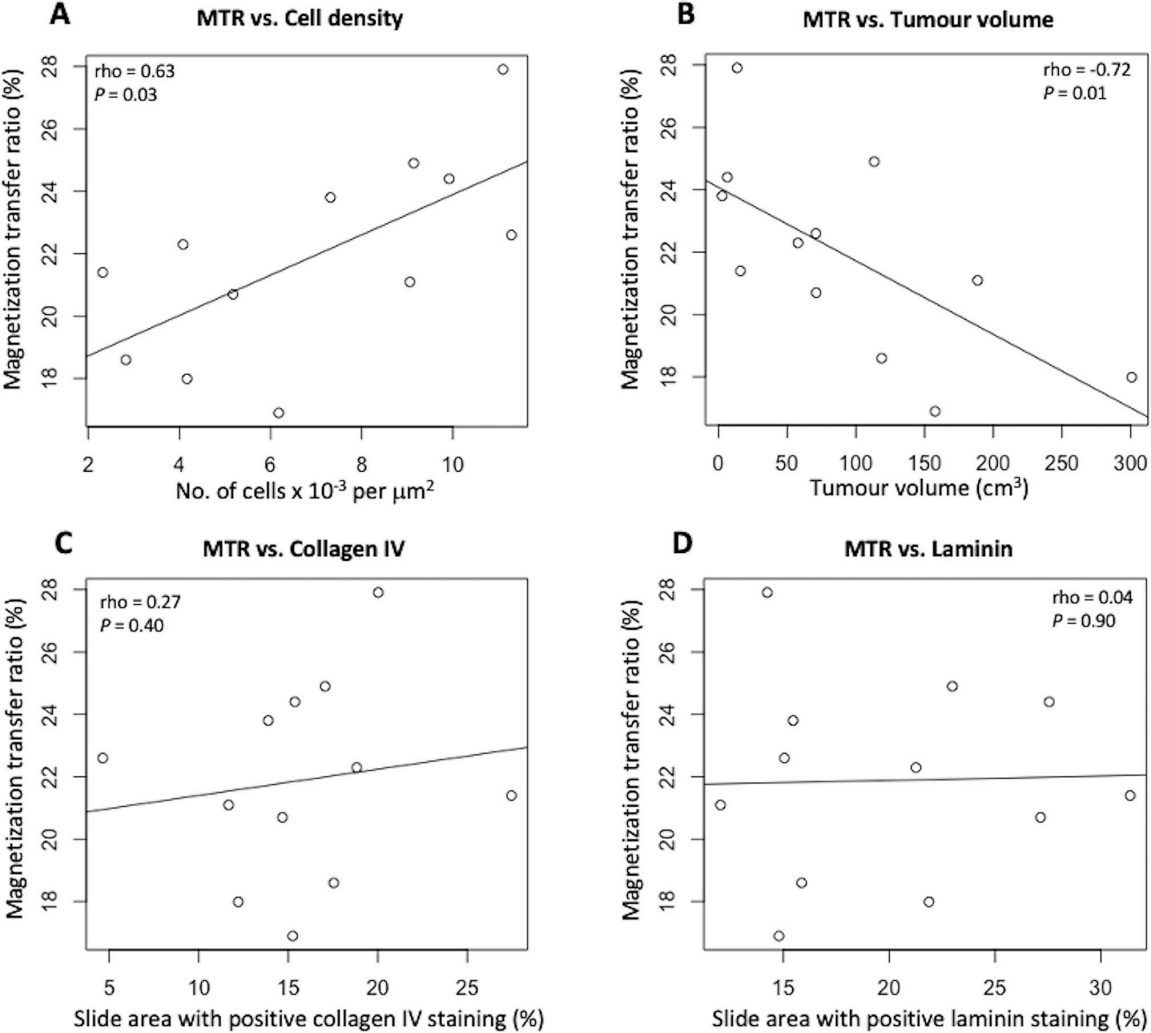
Comparison of cell density, tumour volume and IHC with MT imaging in HGSOC patients. (**A**) MTR compared to cell density; (**B**) MTR compared to tumour volume; (**C**) percentage positive collagen IV tissue area compared to tumour MTR; (**D**) percentage positive laminin tissue area compared to tumour MTR. HGSOC = high-grade serous ovarian cancer; MTR = magnetization transfer ratio.

There was a positive correlation between mean MTR and cell density: Spearman’s ρ = 0.63, *p* = 0.03, and a negative correlation between tumour volume and MTR: ρ = −0.72, *p* = 0.01. Cell density however did not correlate with tumour volume: ρ = 0.24, *p* = 0.44. MTR also did not correlate with collagen IV or laminin: ρ = 0.27, *p* = 0.40 and ρ = 0.04, *p* = 0.90 respectively. Figure 4 graphically demonstrates these correlations with mean MTR.

### Change in MTR with NACT

**Figure 5.**
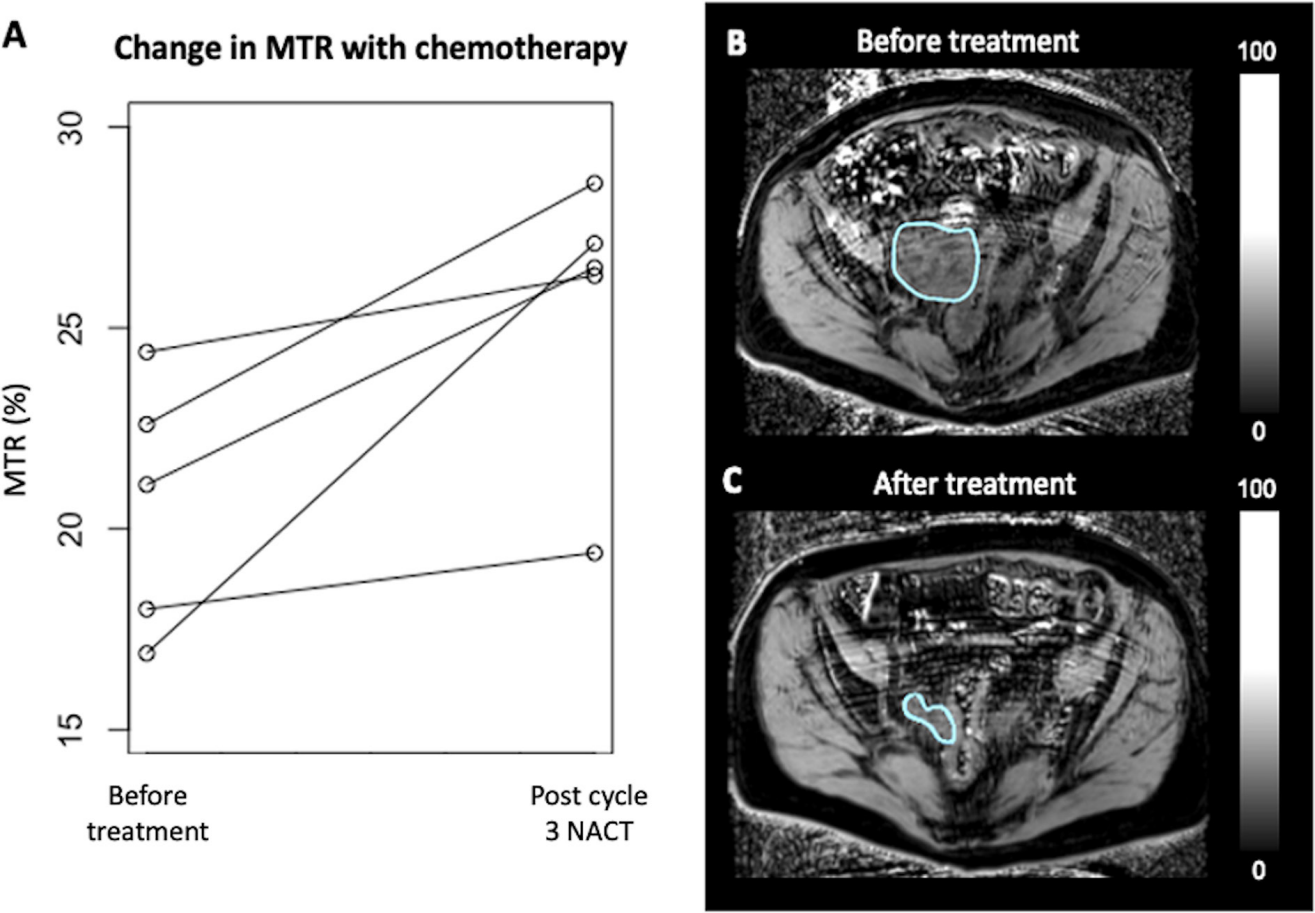
(**A**) Line graphs showing the change in MTR before and after three cycles of neoadjuvant chemotherapy treatment for five patients undergoing repeat imaging; (**B**) example MTR map of a HGSOC tumour before treatment; and (**C**) after treatment. HGSOC = high-grade serous ovarian cancer; MTR = magnetization transfer ratio.

For the five patients who also had imaging after NACT, the mean MTR before treatment was 20.6 ± 3.1% (median 21.1) and the mean MTR after treatment was 25.6 ± 3.4% (median 26.5). An increase in tumour MTR was observed for all individual patient cases. On average, the increase in MTR was 5.0 ± 3.6% between the pre- and post-chemotherapy imaging. Figure 5 shows the change in MTR for each individual patient and a representative example of the pre- and post-treatment MT imaging from one patient. Although there was an increase in the absolute MTR in each case individually, the trend towards increased MTR with treatment marginally failed to reach statistical significance with the Wilcoxon signed-rank test for this small exploratory sample, *p* = 0.06.

## Discussion

In this study, we show that MTR correlates with cell density in treatment-naïve HGSOC, while there is no significant correlation between MTR and the two exclusively extracellular macromolecules that were assessed, collagen IV and laminin. This provides evidence that intracellular macromolecules play an important role in generating the MTR signal in treatment-naïve ovarian cancer, and that MT may therefore serve as an imaging biomarker of cellularity in HGSOC patients. There is potential clinical utility of measuring cellularity while monitoring treatment, as the intended effect of most anticancer drugs is the induction of cell death. This study also showed that MTR correlates negatively with tumour volume. The relationship between volume and MTR could be explained by changes in cell density due to decreased proliferation and increased cell death secondary to a more hypoxic microenvironment in larger tumours.^30,31^ This study also showed a trend towards higher MTR after treatment, but failed to show a statistically significant change given the small subset of patients imaged after NACT as the study was not powered to assess this exploratory end point. However, these data provide preliminary evidence to inform the design of future larger studies to assess the role of MT in assessing treatment response.

The finding of a trend for increased MTR following three cycles of standard-of-care chemotherapy demonstrates the potential of using this technique to monitor treatment effects. An increase in MTR following treatment may be due to decreased tumour volume, or could be caused on a microscopic level by changes in the ratio of the extra- to intracellular volume. There may also be further macromolecular changes that contribute to the MTR in post-treatment tissue which were not specifically evaluated here. For example, previous studies have shown that chemoresistant ovarian cell lines are associated with profibrotic factors,^27^ especially following therapy with taxanes such as paclitaxel which was used within the drug treatment regimen in this study.^28^ Cancer-associated fibroblasts are also known to promote tissue fibrosis following therapy, and this could additionally have increased tumour macromolecular concentrations after treatment in our participants.^29^ Changes on MT imaging after NACT may therefore reflect a combination of both intra- and extracellular macromolecular and microstructural alterations. The standard-of-care NACT treatment in HGSOC of a platinum-based drug combined with a taxane has not changed significantly in over 30 years.^30^ In HGSOC, methods to non-invasively probe the tumour microstructure like MTR could be particularly important for clinical decision-making in NACT patients as alternative treatment options begin to emerge.^31–33^ The detection of microstructural changes in response to standard-of-care NACT can inform management and empower more personalised cancer therapy including switching from ineffective NACT regimens to newer alternative drugs earlier during the treatment course for individual patients based on their distinct tumour characteristics and response which may improve outcomes in HGSOC patients who are non-responders to standard-of-care NACT.

This is the first study to directly compare the measured MT signal with quantitative histology in the high-grade serous subtype of ovarian cancer, including the changes that occur in response to NACT; however, there were several limitations to this study. As with most imaging-pathological correlation studies which rely on tissue acquired at biopsy, a point sample of tissue was compared to the corresponding tumour volume from imaging which could have introduced errors secondary to the spatial heterogeneity of the tumour tissue. The assumption was made that cell density is an indirect measure of the average intracellular macromolecular concentration of the tissue, however, there may have been variations in the intracellular macromolecule concentrations between cells and across tissue samples. Similarly, immunohistochemical measures of the collagen IV and laminin components of the extracellular matrix were taken to be representative of the total extracellular macromolecular pool. Finally, the number of patients imaged after treatment was small due to the exploratory nature of this aspect of the study and there was no post-treatment histological analysis. Despite these limitations, the trend towards higher MTR after treatment established here remains an important finding to inform the design of further larger studies in HGSOC that have ethical approval for multiple time point biopsies.

## Conclusion

These results show that in treatment-naïve HGSOC tissue, the magnetization transfer ratio is weighted towards cell density and therefore intracellular macromolecular concentration. This finding suggests that MT could non-invasively complement focused histological and immunohistochemical analyses of tissue molecular composition and microstructure, by providing information on features such as cellularity across the entire tumour volume. Furthermore, the treatment response element of this study provides evidence that MT imaging may have the potential to detect changes in macromolecular concentrations that occur following NACT although larger studies are needed to validate this. If MT can detect successful response to NACT, then it could be of use in monitoring treatment of HGSOC patients to identify those who may benefit from emerging alternative therapies or to identify microstructural features that can be targeted by novel agents.
